# Toxicological Characterization of the Inorganic and Organic Arsenic Metabolite Thio-DMA^V^ in Cultured Human Lung Cells

**DOI:** 10.1155/2011/373141

**Published:** 2011-10-11

**Authors:** Marc Bartel, Franziska Ebert, Larissa Leffers, Uwe Karst, Tanja Schwerdtle

**Affiliations:** ^1^Institute of Food Chemistry, University of Muenster, Corrensstraße 45, 48149 Muenster, Germany; ^2^Graduate School of Chemistry, University of Muenster, 48149 Muenster, Germany; ^3^Institute of Inorganic and Analytical Chemistry, University of Muenster, Corrensstraße 30, 48149 Muenster, Germany

## Abstract

We synthesised and toxicologically characterised the arsenic metabolite thiodimethylarsinic acid (thio-DMA^V^). Successful synthesis of highly pure thio-DMA^V^ was confirmed by state-of-the-art analytical techniques including ^1^H-NMR, HPLC-FTMS, and HPLC-ICPMS. Toxicological characterization was carried out in comparison to arsenite and its well-known trivalent and pentavalent methylated metabolites. It comprised cellular bioavailability as well as different cytotoxicity and genotoxicity end points in cultured human A549 lung cells. Of all arsenicals investigated, thio-DMA^V^ exerted the strongest cytotoxicity. Moreover, thio-DMA^V^ did not induce DNA strand breaks and an increased induction of both micronuclei and multinucleated cells occurred only at beginning cytotoxic concentrations, indicating that thio-DMA^V^ does not act via a genotoxic mode of action. Finally, to assess potential implications of thio-DMA^V^ for human health, further mechanistic studies are urgently necessary to identify the toxic mode of action of this highly toxic, unusual pentavalent organic arsenical.

## 1. Introduction

Inorganic arsenic is a well-documented human carcinogen (IARC, Group 1) causing tumors in the lung, skin, and bladder [1, 2]. However, the underlying molecular mechanisms of inorganic arsenic-induced carcinogenicity are still to be elucidated, especially since inorganic arsenic, unlike other classical chemical carcinogens, does neither induce direct DNA damage nor mutagenicity at exposure-relevant concentrations [3]. Besides the contribution of its metabolism, a variety of further potential mechanisms are discussed, including the induction of genetic damage via oxidative mechanisms [4–6], epigenetic dysregulation [7], and interaction with the cellular DNA damage response and DNA repair [8], resulting in comutagenic and cocarcinogenic effects [9]. 

For the general population, human diet is the primary source of both total arsenic and inorganic arsenic intake. The European Food Safety Authority (EFSA) Panel on Contaminants in the Food Chain and the Joint Food and Agriculture Organization/World Health Organisation (FAO/WHO) Expert Committee on Food Additives (JECFA) have concluded in their recent scientific opinions on arsenic that risks to human health related to the presence of inorganic arsenic in food cannot be excluded. In 2010, the JECFA withdrew the previous provisional tolerable weekly intake (PTWI) [9–11]. Furthermore, the EFSA Panel emphasized the necessity for toxicological characterization of seafood and fish-related organic arsenicals, including arsenosugars and arsenolipids, for which to date no toxicological data exist [9]. In contrast to arsenobetaine, which is the main arsenic compound in fish, but is not metabolized by humans, arsenosugars and arsenolipids are extensively biotransformed to a multitude of arsenic metabolites [12, 13]. Some of these compounds are believed to be highly toxic and thus it cannot be excluded that seafood and fish-related organic arsenic species present risks to human health.

Regarding *in vitro* toxicity of the well-known and partly toxicologically characterised human inorganic arsenic metabolites monomethylarsinous (MMA^III^), dimethylarsinous (DMA^III^), monomethylarsonic (MMA^V^), and dimethylarsinic (DMA^V^) acid, the trivalent metabolites exert stronger cytotoxicity, as well as direct and indirect genotoxicity as compared to arsenite [14–21] in most cellular and subcellular test systems. Therefore, trivalent methylated arsenicals are generally believed to strongly contribute to inorganic arsenic-induced genotoxicity and, most likely, carcinogenicity. 

Thiodimethylarsinic acid (thio-DMA^V^, [(CH_3_)_2_As(S)OH], also named dimethylmonothio-arsinic acid, DMMTA^V^ or DMTA^V^) is the pentavalent sulfur analogue of DMA^V^ and a metabolite of organic as well as inorganic arsenicals. The first identification of thio-DMA^V^ as a mammalian arsenic metabolite was obtained in urine and wool extract from a sheep naturally consuming large amounts of arsenosugars through seaweed [22]. In this paper, the group of Feldmann also discussed the serious problem that thio-DMA^V^ may have been misidentified as DMA^III^ in human urine samples before and, therefore, might have escaped detection in many samples so far [22, 23]. Indeed, thio-DMA^V^ has later been identified in human urine after exposure towards arsenosugars as well as inorganic arsenic-contaminated drinking water [12, 13, 23]. In a recent study investigating the arsenic metabolites in urine samples of 75 inorganic arsenic-exposed women in Bangladesh, thio-DMA^V^ has been shown to be a common metabolite, being detected in 44% of the samples [23]. Furthermore, thio-DMA^V^ might also directly occur in food, which has been postulated before for rice [24]. 

Probably because thio-DMA^V^ is not commercially available, in the literature no *in vivo* toxicity studies for thio-DMA^V^ (except for toxicokinetic studies) and only few *in vitro* toxicity studies exist. Nevertheless, these few studies point to a quite strong cellular toxicity of thio-DMA^V^ in mammalian cells in culture. Thus, in most studies, thio-DMA^V^ showed much higher cytotoxicity as compared to MMA^V^ and/or DMA^V^ [23, 25] and comparable effects to trivalent arsenicals [26, 27]. In some studies, thio-DMA^V^ even exerted stronger cytotoxicity as compared to arsenite [27–29]. Moreover, Ochi et al. provided evidence for a genotoxic potential of thio-DMA^V^ in cultured hamster cells [25], whereas no detailed data exist regarding the genotoxicity of thio-DMA^V^ in human cells.

The aim of the present study was to further investigate the toxicity of thio-DMA^V^ in cultured human A549 lung cells. Therefore, we synthesised and analytically characterised highly pure dimethylthioarsinic anhydride, which in aqueous solution immediately forms thio-DMA^V^. Subsequently, cytotoxicity, cellular uptake, as well as for the first time genotoxicity at the DNA and chromosomal levels were examined in cultured human cells, while comparing effects of thio-DMA^V^ with effects of arsenite, MMA^III^, DMA^III^, MMA^V^, and DMA^V^.

## 2. Materials and Methods

### 2.1. Caution

Inorganic arsenic is classified as a human carcinogen. The following chemicals are hazardous and should be handled with care: sodium arsenite, methyloxoarsine (precursor to MMA^III^), iododimethylarsine (precursor to DMA^III^), dimethylthioarsenic anhydride (precursor to thio-DMA^V^), MMA^V^, and DMA^V^.

### 2.2. Materials

Dulbecco's modified Eagle medium (DMEM), foetal calf serum (FCS), trypsin, and penicillin-streptomycin solutions were obtained from Sigma-Aldrich (Steinheim, Germany). The culture dishes were supplied by Biochrom (Berlin, Germany). Sodium(meta)arsenite (≥99% purity) and Alcian Blue were purchased from Fluka Biochemika (Buchs, Germany). Methyloxoarsine (CH_3_As^III^O, ≥99% purity) and iododimethylarsine [(CH_3_)_2_As^III^I, ≥99% purity] (storage at −80°C) were kindly provided by Professor Dr. W. Cullen (University of British Columbia, Vancouver, Canada). DMA^V^ (≥99% purity) and MMA^V^ (99% purity) were purchased from Sigma-Aldrich (Steinheim, Germany). A549 cells (CCL-185) were obtained from the American Type Culture Collection (Bethesda, MD, USA). 

Giemsa dye and acridine orange were bought from Roth (Karlsruhe, Germany). The ICPMS elemental standard (As, 1 mg/L) was purchased from SPETEC (Erding, Germany). Hydrogen peroxide solution (30%, Suprapur) and nitric acid (65%, Suprapur) were products of Merck (Darmstadt, Germany). Triton X-100 was bought from Pierce (Oud-Beijerland, The Netherlands), hydroxyapatite (high resolution) from Calbiochem (Bad Soden, Germany) and Hoechst 33258 from Merck (Darmstadt, Germany). All other proanalysis chemicals were obtained from Sigma-Aldrich (Steinheim, Germany) or Merck (Darmstadt, Germany).

### 2.3. Synthesis of Dimethylthioarsinic Anhydride

Dimethylthioarsinic anhydride, which dissociates in water to thio-DMA^V^, was synthesized according to Fricke et al. [30]. Briefly, DMA^V^ was dissolved in ethanol (30%) and hydrogen sulfide was bubbled into the solution and stirred over night. After removing the solvent, the residue was extracted with chloroform/water (3 : 1) and the chloroform layer was washed with water to remove the remaining water-soluble arsenic compounds. Finally, the solvent was removed and dimethylthioarsinic anhydride was recrystallized from methanol/hexane.

### 2.4. Analysis and Purity Control of Dimethylthioarsinic Anhydride

HPLC-FTMS (Thermo Accela, Thermo LTQ Orbitrap XL), HPLC-ICPMS (Shimadzu LC-10, Perkin Elmer ELAN 6000), and electrothermal AAS (Perkin Elmer, AAnalyst 600) were applied for identification and quantification as well as to obtain information about purity of the arsenic species. Thio-DMA^V^ solutions in water were prepared directly for each experiment; in order to compensate for sensitivity differences, the sample used for HPLC-FTMS (20 mg/L) was diluted by a factor of 400 for HPLC-ICPMS analysis. Briefly, for chromatographic separation, PTFE autosampler vials, a reversed-phase column (Waters Atlantis T3, 2.1 × 150 mm, 5 *μ*m), and the eluent 13.2 mM ammonium acetate in water/10% methanol (pH 4.6) [30] were used. The flow rate was 0.3 mL/min. The m/z range for HPLC-FTMS analysis was set from m/z 80 to 1000. Fragmentation experiments were carried out with collisionally induced dissociation (CID) using normalized collision energy, and data analysis was performed using Xcalibur software. For quantification of arsenic species by HPLC-ICPMS, chromatographic data were collected by monitoring m/z 75 (As and ^40^Ar^35^Cl) and 77 (^40^Ar^37^Cl) with 100 ms dwell time. The results of the chromatography were analyzed with the data analysis software from OriginLab. Quantification of total As in thio-DMA^V^ solutions was carried out by electrothermal AAS, applying an ICPMS elemental standard.

Furthermore, ^1^H-NMR spectroscopy (Bruker DCX-400, 400 MHz) was used to obtain additional purity information. Arsenic species were dissolved in deuterium oxide (D_2_O), and the chemical shift values were observed for structural information. The obtained results were evaluated with the NMR data software MestReNova (Mestrelab Research) and compared with data from the literature.

### 2.5. Cell Culture and Incubation with the Arsenicals

Since the lung is an important target organ for inorganic arsenic-induced carcinogenicity, human A549 epithelial lung adenocarcinoma cells were used as *in vitro* model system. A549 cells were grown in culture dishes as monolayer in DMEM containing 10% FCS, 100 U penicillin/mL, and 100 *μ*g streptomycin/mL. The cultures were incubated at 37°C with 5% CO_2_ in air and 100% humidity. 

Arsenical stock solutions were prepared in sterile deionised water. All stock solutions were prepared shortly before each experiment, among others to prevent oxidation of trivalent arsenicals. Logarithmically growing A549 cells were incubated with the arsenicals for 1 h or 24 h as described for the respective experiments.

### 2.6. Cytotoxicity Testing of Thio-DMA^V^

The cytotoxicity of thio-DMA^V^ was elucidated by quantifying its effect on cell number and colony forming ability. Cell number and colony forming ability testing were exactly performed as described before for inorganic arsenic, MMA^III^, DMA^III^, MMA^V^, and DMA^V^ [31]. Briefly, after 24 h of incubation with the respective arsenicals, cells were washed with phosphate buffered saline (PBS) and trypsinized. Subsequently, cell number and cell volume were measured by an automatic cell counter (Casy-1, Roche Innovatis AG, Bielefeld, Germany). These measurements are based on noninvasive (dye-free) electrical current exclusion with signal evaluation via pulse area analysis. To assess the impact of thio-DMA^V^ on colony forming ability of A549 cells, after cell counting of each sample, 300 cells/dish were seeded. After 7 days of incubation, colonies were fixed with ethanol, stained with Giemsa (25% in ethanol), counted and calculated as percent of control.

### 2.7. Cellular Bioavailability

To compare cellular bioavailability of thio-DMA^V^ with cellular bioavailability of inorganic arsenic and its related methylated metabolites, cellular bioavailability studies were carried out by exactly the same protocol as previously reported [31]. Briefly, logarithmically growing cells (1∗10^6^) were exposed to thio-DMA^V^ for 24 h, trypsinized, collected by centrifugation, washed with ice-cold PBS, and cell number as well as cell volume were measured by an automatic cell counter in each sample as described before. After incubation with the ashing mixture (65% HNO_3_/30% H_2_O_2_ (1/1, v/v)) at 95°C for at least 12 h, samples were diluted with bidistilled water, and arsenic was measured by electrothermal atomic absorption spectrometry (AAnalyst 600, Perkin Elmer).

### 2.8. Determination of DNA Strand Breaks

DNA strand breaks were quantified by alkaline unwinding as described previously [32]. Briefly, 1∗10^5^ cells were seeded, allowed to attach for 24 h and incubated with thio-DMA^V^ for 1 or 24 h. Subsequently, the medium was removed, cells were washed with PBS and an alkaline solution containing 0.03 M NaOH, 0.02 M Na_2_HPO_4_, and 0.9 M NaCl was added. After neutralisation and sonication, separation of single- and double-stranded DNA was performed on 0.5 mL hydroxyapatite columns at 60°C. Single- and double-stranded DNA were eluted with 1.5 mL of 0.15 M and 0.35 M potassium phosphate buffer, respectively. The DNA content of both fractions was determined by adding Hoechst 33258 dye to a final concentration of 7.5∗10^−7^ M to 1 mL of each sample and measuring the fluorescence with a microtiter fluorescence reader (FLUOstar Optima, BMG Labtechnologies, Jena, Germany) at an excitation wavelength of 360 nm and an emission wavelength of 455 nm. DNA strand breaks were quantified by calibration with X-rays as described previously [33].

### 2.9. Formation of Micronuclei and Multinucleated Cells

By the early 1990s, the micronucleus assay was shown to be suitable to investigate arsenic-induced chromosomal alterations as a biological marker of its genotoxicity [34]. In recent years, the *in vitro *micronucleus assay has become an attractive tool for genotoxicity testing in general [35]. Therefore, this endpoint has been (and is) strongly used to characterize the genotoxic potential of arsenicals in epidemiological studies [36, 37] as well as in cultured mammalian cells (e.g., [38]). To investigate the induction of micronuclei and multinucleated cells, in this study A549 cells were seeded in 6-well plates on Alcian blue coated glass coverslips. After 24 h, cells were incubated with the respective arsenicals for 24 h, fixed with an ice-cold fixation solution (90% methanol/10% PBS, −20°C) for 10 min, dried in the air at room temperature, stained with acridine orange (125 mg/L in PBS) for 10 s, and finally analyzed by fluorescence microscopy. Per coverslip, at least 1000 cells were counted and categorized in mononucleated, binucleated, and multinucleated cells as well as cells with and without micronuclei.

## 3. Results

### 3.1. Synthesis, Analysis and Purity Control of Dimethylthioarsinic Anhydride

**Figure 1:**
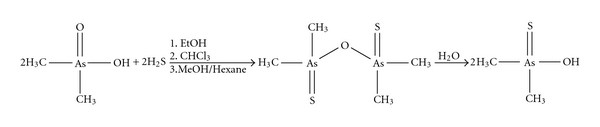
Scheme of the synthesis of dimethylthioarsinic anhydride and its conversion into the acid form thio-DMA^V^ in water.

Colorless, highly pure dimethylthioarsinic anhydride crystals were obtained by the reaction of DMA^V^ with H_2_S in ethanol, followed by extraction with chloroform and recrystallisation from methanol/hexane (Figure 1). After dissolving dimethylthioarsinic anhydride in water, formation of thio-DMA^V^ was analysed by means of hyphenated techniques and ^1^H-NMR.

**Figure 2:**
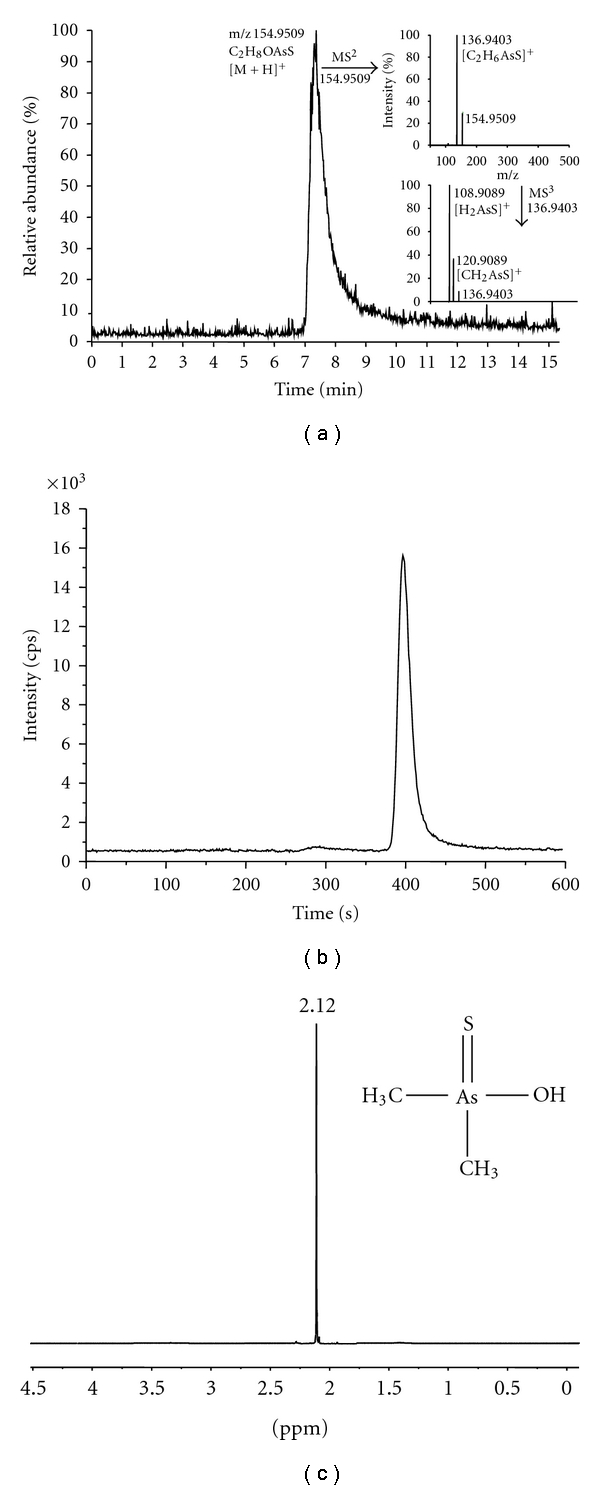
Analytical characterization of thio-DMA^V^. (a): HPLC-FTMS total ion chromatogram including fragmentation spectra of the parent ion m/z 154.9509 [C_2_H_8_OAsS]^+^; (b): HPLC-ICPMS chromatogram selectively monitoring m/z 75 (As); (c): ^1^H-NMR spectrum of thio-DMA^V^ in D_2_O.

The mass spectrometric data and the ^1^H-NMR results confirmed the conversion of the synthesized anhydride in water into the acid form thio-DMA^V^. Structural elucidation of thio-DMA^V^ was performed by HPLC-FTMS analysis and by determination of the exact mass. The calculated mass of the acid form is m/z 154.9512 [M+H]^+^and the detected mass was m/z 154.9509. Fragmentation experiments with collisionally induced dissociation of the parent ion m/z 154.95 [C_2_H_8_OAsS]^+^ gave a fragment ion of m/z 136.9403 [C_2_H_6_AsS]^+^ and further fragmentation led to m/z 108.9089 [H_2_AsS]^+^. The corresponding HPLC-FTMS total ion chromatogram (Figure 2(a)) demonstrates thio-DMA^V^ as a protonated molecular ion with a retention time of 7.3 min, whereas neither starting material nor further reaction products were detected. 

HPLC-ICPMS analyses further verified the purity of thio-DMA^V^ by retention time matching of known arsenic species, including DMA^V^ as the starting material. Thus, after dissolving thio-DMA^V^ in water, HPLC-ICPMS chromatograms showed only one compound (Figure 2(b)). Under additional consideration of the quantification of thio-DMA^V^ by electrothermal AAS, the purity of dimethylthioarsinic anhydride was assessed to be ≥98%. 

^1^H-NMR measurements of thio-DMA^V^ (Figure 2(c)) in D_2_O resulted in a chemical shift value of 2.12 ppm which is similar to the value of 2.11 ppm reported by Fricke et al. [30]. DMA^V^ showed a chemical shift of 1.98, the range of which is consistent with a pentavalent arsenical. The ^1^H-NMR data of thio-DMA^V^ showed no impurities, and the desired compound was obtained in analytically pure form based on ^1^H-NMR spectroscopy.

### 3.2. Cytotoxicity of Thio-DMA^V^

**Figure 3:**
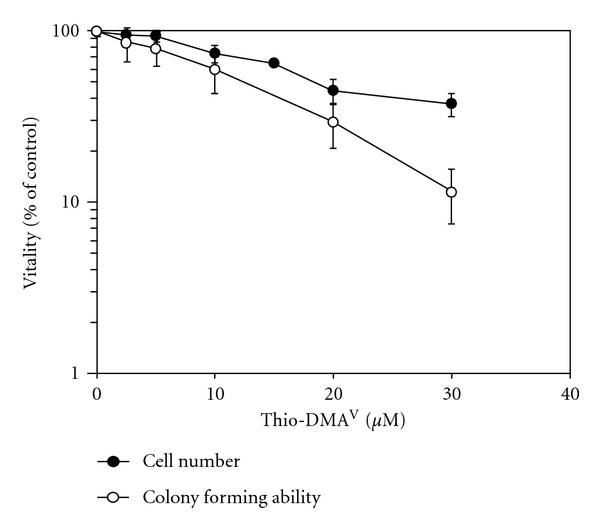
Cytotoxicity of thio-DMA^V^ in A549 cells after 24 h incubation. Cytotoxicity was determined by a decrease in cell number (closed symbols) and effects on colony forming ability (open symbols). The data represent mean values of at least six determinations ± SD.

**Figure 4:**
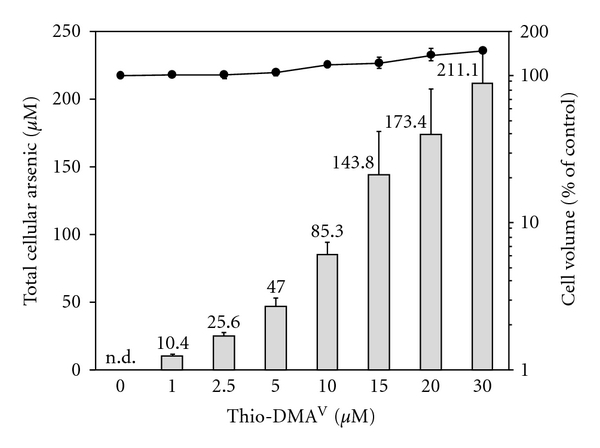
Cellular bioavailability of arsenic in A549 cells after 24 h incubation with thio-DMA^V^. Logarithmically growing A549 cells were treated with thio-DMA^V^ for 24 h, trypsinized, and cell number as well as cell volume was determined. Finally, arsenic was quantified by electrothermal atomic absorption spectroscopy. Shown are mean values of at least six independent determinations +/± SD; n.d.: below detection limit.

Cytotoxicity of thio-DMA^V^ was determined by investigating its effects on cell number and colony forming ability (Figure 3) after 24 h incubation. The cell volume (Figure 4) was determined as well, however, principally to calculate cellular arsenic concentrations later on. Regarding both endpoints, cell number and colony forming ability, thio-DMA^V^ exerted higher cytotoxicity as compared to arsenite and especially to the pentavalent methylated metabolites MMA^V^ and DMA^V^, whereas effects were about twofold lower as compared to MMA^III^ and DMA^III^ (Table 1). Thio-DMA^V^ affected colony forming ability stronger as compared to cell number, which is comparable to the trivalent methylated metabolites. In case of arsenite, MMA^V^ and DMA^V^, both cytotoxicity endpoints showed similar sensitivity.

### 3.3. Cellular Bioavailability of Thio-DMA^V^

To assess cellular bioavailability in A549 cells and to correlate cellular toxicity of thio-DMA^V^ with cellular arsenic content, cellular arsenic concentrations were determined after 24 h incubation by electrothermal atomic absorption spectrometry. 

Comparing extracellular and intracellular arsenic concentrations, a 9-10-fold accumulation was observed in cells incubated with up to 15 *μ*M thio-DMA^V^ (Figure 4). Thio-DMA^V^ showed no significant effects on cell volumes (Figure 4) at noncytotoxic concentrations, but increased cell volumes in case of cytotoxic concentrations (≥10 *μ*M) by up to 44%. Mean (±SD) volumes of nonincubated control cells were 2.68  (±0.14)∗10^−12^ L. 

Interestingly, the concentration of cellular arsenic strongly correlated with the cytotoxicity of thio-DMA^V^, resulting in a correlation coefficient of −0.986 (cell number) or −0.998 (colony forming ability), respectively.

### 3.4. Induction of DNA Strand Breaks by Thio-DMA^V^

**Figure 5:**
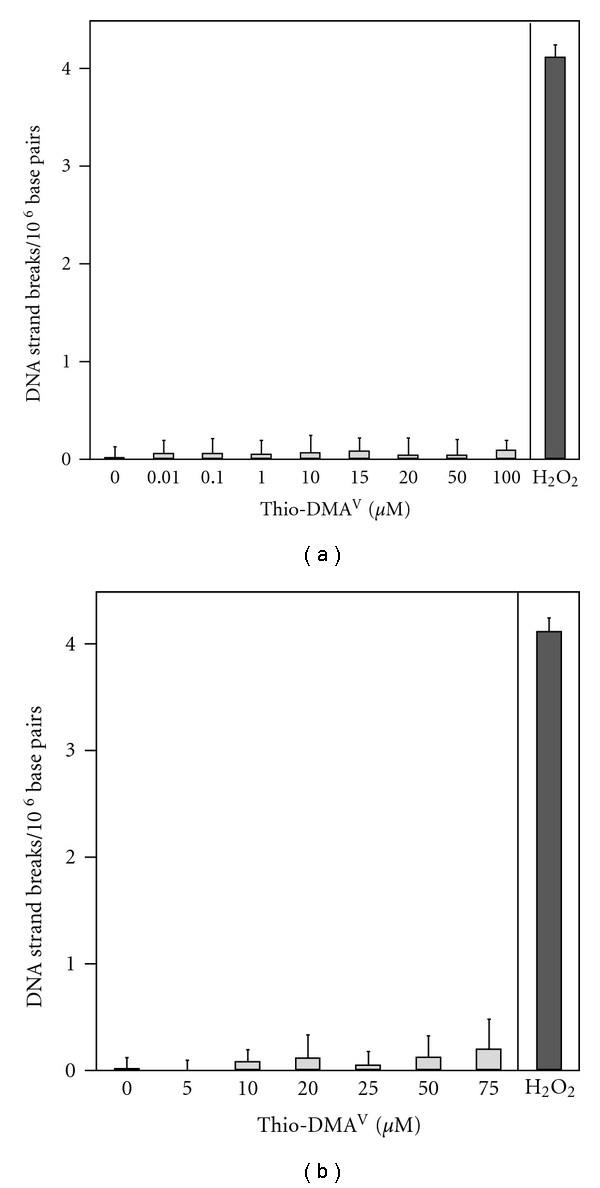
DNA strand break formation after 1 h (a) and 24 h (b) incubation with thio-DMA^V^ in A549 cells. DNA strand breaks were quantified by alkaline unwinding; 5 min incubation with 75 *μ*M H_2_O_2_ served as positive control. Shown are mean values of at least six determinations + SD.

A possible generation of DNA strand breaks by thio-DMA^V^ was investigated in A549 cells after short-term (1 h) and long-term (24 h) incubation, applying the alkaline unwinding technique. Up to high, already cytotoxic thio-DMA^V^ concentrations both after 1 h and after 24 h incubation, no significant induction of DNA strand breaks was observed (Figures 5(a) and 5(b)).

### 3.5. Formation of Micronuclei and Multinucleated Cells by the Arsenicals

The two basic mechanisms leading to the onset of micronuclei are disturbance of the chromosome segregation machinery and chromosome breakage. Thus, in somatic cells, micronuclei can only occur after mitotic division, and in the cytokinesis-block micronucleus assay (CBMN), which is based on cytokinesis inhibition by cytochalasin B, cell proliferation and thereby mitosis are generally controlled by a scoring of mono- and binucleated cells [35]. However, our first CBMN studies indicated that several arsenicals interact with actin and/or the effect of cytochalasin B (data not shown). To assess the induction of micronuclei by the arsenicals, we omitted the application of cytochalasin B. To ensure mitosis, we controlled cell proliferation by means of cell number quantification and chose an incubation time of 24 h, which is equivalent to 1.25 cell cycles of the A549 lung cells. This incubation time was previously used to examine cellular toxicity of arsenicals in A549 cells [31] and, therefore, opens the possibility to compare results. Furthermore, this protocol allows the proper quantification of the formation of multinucleated cells by the arsenicals at the same time. 

**Figure 6:**
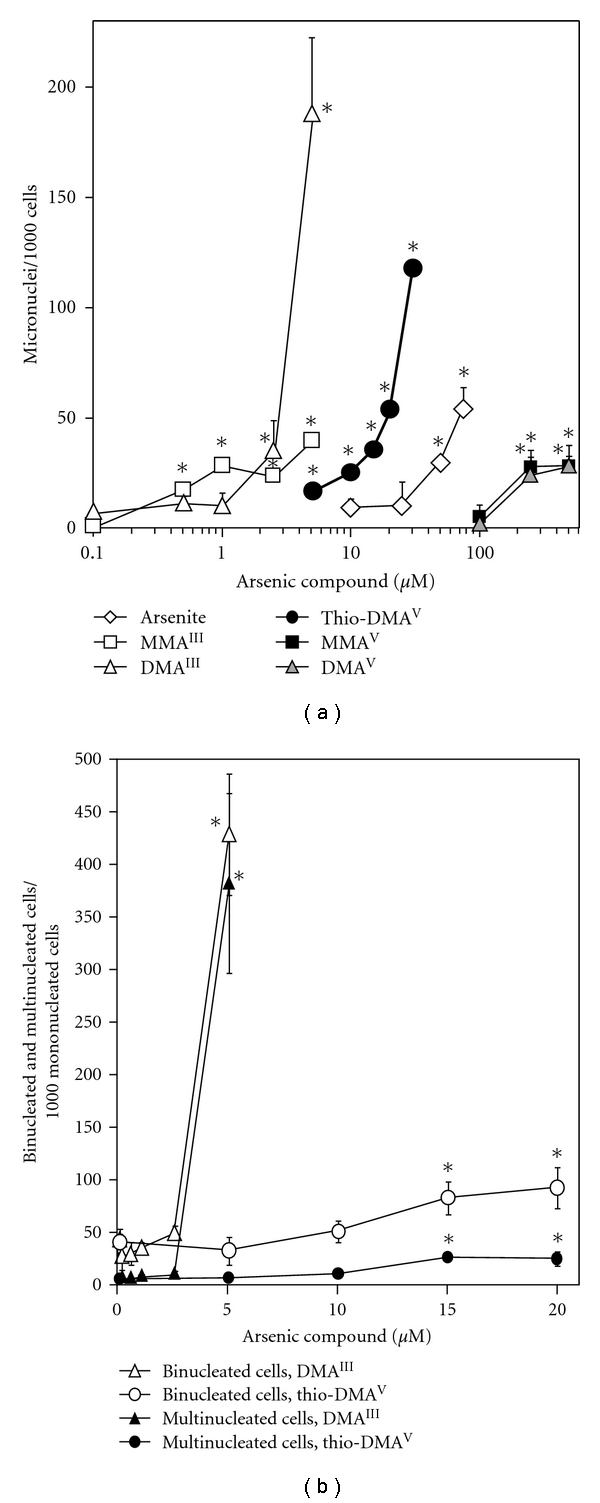
Induction of micronuclei (a), binucleated and multinucleated cells (b) after 24 h incubation of A549 cells. After 24 h in culture, logarithmically growing A549 cells were treated with arsenite, MMA^III^, DMA^III^, thio-DMA^V^, MMA^V^, or DMA^V^ for 24 h; cells were fixed, stained and finally analysed by fluorescence microscopy as described in Materials and Methods. (a): additionally induced micronuclei, micronuclei level in control was 27 ± 3; (a, b): shown are mean values of at least three independent determinations + SD; statistically significant different from nonarsenic exposed controls: *P < 0.01 as determined by Student's *t*-test.

At noncytotoxic to beginning slightly cytotoxic concentrations, MMA^III^ (0.5, 1 *μ*M), thio-DMA^V^ (5 *μ*M), MMA^V^ (250 *μ*M), and DMA^V^ (250 *μ*M) induced a small, but significant number of around 20 micronuclei (Figure 6(a)). At higher, already cytotoxic arsenic species concentrations, micronuclei formation increased and became also significant in case of arsenite (≥50 *μ*M). At highly cytotoxic concentrations, 5 *μ*M DMA^III^ and 30 *μ*M thio-DMA^V^ showed strongest effects, inducing 188 ± 34.5 and 118 ± 4.6 micronuclei, respectively. 

Moreover, thio-DMA^V^ and especially DMA^III^ increased the formation of multinucleated cells and the occurrence of binucleated cells in comparison to untreated control cells (Figure 6(b)). However, significant effects were restricted to cytotoxic concentrations. For all other applied arsenicals, no significant increased occurrence of bi- and multinucleated cells was observed.

## 4. Discussion

The data presented in this study provide further evidence for the strong cellular toxicity of the recently identified arsenic metabolite thio-DMA^V^ in human cells. 

In the applied human lung cells, cytotoxicity of thio-DMA^V^ strongly correlates with its cellular bioavailability. For other arsenic species, a similar correlation has been reported in A549 cells [31] as well as in human urothelial (UROtsa) and hepatic (HepG2) cells [39] before. 

When comparing the respective arsenic incubation concentrations, thio-DMA^V^ exerts higher cytotoxicity than arsenite, whereas effects are lower as compared to MMA^III^ and DMA^III^. When additionally taking into account the cellular bioavailability of the arsenicals, among all arsenicals, applied thio-DMA^V^ shows the highest cytotoxicity in A549 cells. For instance, 30% reduction in cell number occurred after 24 h incubation with 5 *μ*M DMA^III^, which is related to 237 ± 38.2 *μ*M cellular arsenic [31]. 12.1 *μ*M thio-DMA^V^ caused a similar reduction in cell number; however, it corresponds to a cellular arsenic concentration of 115 ± 9.4 *μ*M. Thus, a similar cytotoxic effect is achieved at twofold lower cellular arsenic concentrations. In summary, referring to the extracellular incubation concentrations, in A549 human lung cells the arsenicals follow the cytotoxic order: DMA^III^ > MMA^III^ > thio-DMA^V^≫ arsenite ≫ MMA^V^ ~ DMA^V^. Taking into account the cellular uptake of the arsenic species and thereby referring to the effective cellular arsenic concentrations, the cytotoxic order switches to thio-DMA^V^~ arsenite ~ MMA^III^ > DMA^III^ ≫ MMA^V^ ~ DMA^V^. This is somehow contrary to the study by Naranmandura et al. [26], where at the respective IC50 concentrations, cellular thio-DMA^V^ uptake was higher as compared to DMA^III^ and arsenite uptake. This different outcome might be due to the different cell systems applied, but most likely results from the different cytotoxicity endpoints investigated. By using the MTT test, Naranmandura et al. used a cellular metabolism-related cytotoxicity endpoint, which quantifies the impact of the arsenicals on the activity of cellular dehydrogenases. In contrast, in this study we quantified cell number, which comprises cell death and proliferation inhibition by the arsenicals. Moreover, colony forming ability, which is generally considered as benchmark long term-cytotoxicity assay for directly not acute cytotoxic compounds, was applied as second cytotoxicity endpoint. Very interestingly, thio-DMA^V^ exerts stronger cytotoxicity regarding the endpoint colony forming ability, which points to an indirect mode of toxic action. This has similarly been shown before for the trivalent methylated metabolites [31]. 

In contrast to all other methylated arsenic metabolites [18], in A549 cells thio-DMA^V^ showed no generation of DNA strand breaks up to high cytotoxic concentrations. This is also in line with the fact that thio-DMA^V^ did not significantly increase reactive oxygen species level in A549 cells (as assessed by DCFDA fluorescence) up to high cytotoxic concentrations (data not shown). This is in contrast to the postulated, reactive oxygen species-mediated toxic mode of action of thio-DMA^V^ [28, 40]. Accordingly thio-DMA^V^, as well as DMA^III^ and arsenite, exerted strong genotoxicity on the chromosomal level only at cytotoxic concentrations. Thus, in the present study, MMA^III^ is the only arsenical-inducing micronuclei at noncytotoxic, exposure-relevant concentrations starting at 0.5 *μ*M. Micronuclei formation in A549 cells results at least partly from the earlier observed induction of DNA damage by 0.5 *μ*M MMA^III^ [18].

For DMA^III^, micronuclei induction has been shown before in CHO cells [38] and is discussed to be due to both aneugenic and clastogenic effects of DMA^III^. In SHE (Syrian hamster embryo) cells after 24 h incubation, 20 *μ*M thio-DMA^V^ induced chromosome structural aberrations including chromatid gaps, chromatid break and chromatid changes [25]. This fits nicely to earlier data by Kuroda et al. in V79 Chinese hamster lung cells: here, the unknown microbial metabolite of DMA^V^, which is nowadays strongly discussed to be thio-DMA^V^, induced chromosomal aberrations as well as sister chromatid exchange, mitotic arrest, and tetraploids [41]. In this study, thio-DMA^V^ and especially DMA^III^ additionally increased the formation of multinucleated and binucleated cells, which most probably results from spindle abnormalities induced by these arsenic species [25, 42]. Furthermore, the increased formation of binucleated cells indicates an inhibitory effect of DMA^III^ and thio-DMA^V^ on cytokinesis. Accordingly, in the same concentration range, both arsenicals caused a G2/M cell cycle phase arrest after 24 h incubation in A549 cells (data not shown). For thio-DMA, this has already been shown before in human HepG2 hepatocarcinoma cells [25] and A431 epidermoid carcinoma cells [26]. 

When rating the formation of micronuclei, micronucleated and binucleated cells, it once again has to be clearly stated that all these effects were restricted to high concentrations of arsenite, thio-DMA^V^, and DMA^III^. Strong effects were observed exclusively for DMA^III^, with a sevenfold increase in micronuclei induction, a tenfold increased occurrence of binucleated cells, and an 80-fold increase in multinucleated cells after 24 h incubation with 5 *μ*M DMA^III^. Thus, in case of DMA^III^, these effects most likely trigger DMA^III^ cytotoxicity, especially regarding the endpoint colony forming ability. This is unlikely for thio-DMA^V^ and even more unlikely for arsenite. 

In summary, thio-DMA^V^ seems to exert its high cellular toxicity by a different mode of action than arsenite, MMA^III^, and DMA^III^. Our data strongly indicate that in human A549 lung cells, thio-DMA^V^ does not act via a genotoxic mode of action. Nevertheless, to assess the role of thio-DMA^V^ in inorganic arsenic-induced carcinogenicity, to date still too little is known about thio-DMA^V^. This is particularly valid as thio-DMA^V^ is a human metabolite not only of inorganic arsenic but also of seafood related organic arsenicals, which indicates that further mechanistic studies are urgently needed to identify its toxic mode of action and finally assess the potential implications for human health.

## Figures and Tables

**Table 1:** Cytotoxic effects of the arsenicals in A549 cells after 24 h incubation. Shown are IC70 values for the endpoints cell number and colony forming ability. In case of arsenite, MMA^III^, DMA^III^, MMA^V^, and DMA^V^ IC70 values were generated from the data originally published in Ebert et al. 2011 [31]; IC70 values represent the respective inhibitory concentrations of the compounds that are required for 30% reduction of cell number or colony forming ability *in vitro*.

Arsenic species	IC70 (cell number)	IC70 (colony forming ability)
Arsenite	57.2 *μ*M	58.8 *μ*M
MMA^III^	5.6 *μ*M	3.8 *μ*M
DMA^III^	5.1 *μ*M	3.2 *μ*M
Thio-DMA^V^	12.1 *μ*M	7.2 *μ*M
MMA^V^	>500 *μ*M	>500 *μ*M
DMA^V^	>500 *μ*M	>500 *μ*M
